# Neoadjuvant camrelizumab in combination with chemotherapy followed by sequential radical surgery for locally advanced esophageal squamous cell carcinoma: postoperative pathological remission characteristics and influencing factors

**DOI:** 10.3389/fonc.2026.1789575

**Published:** 2026-05-15

**Authors:** Jiaxin Pei, Ziyi Xiao, Yushu Deng, Yikai Han, Mengmeng Yang, Feng Wang, Taiying Lu

**Affiliations:** Department of Oncology, The First Affiliated Hospital of Zhengzhou University, Zhengzhou, Henan, China

**Keywords:** camrelizumab, esophageal squamous cell carcinoma, neoadjuvant therapy, pathological response, peripheral blood inflammatory markers, prognosis

## Abstract

**Background:**

Neoadjuvant camrelizumab combined with chemotherapy has shown favorable efficacy and safety profiles in the treatment of locally advanced esophageal squamous cell carcinoma (LA-ESCC). This study focused on the clinical data of patients treated with camrelizumab plus chemotherapy before and after neoadjuvant treatment, with the aims of exploring predictive markers for pathological response after radical resection, systematically verifying the safety of this regimen, and investigating indicators associated with long-term prognosis of patients.

**Methods:**

A retrospective analysis was conducted on patients with histologically confirmed LA-ESCC who were treated at the First Affiliated Hospital of Zhengzhou University between November 2019 and September 2024. Peripheral blood inflammatory and nutritional parameters, adverse events, and perioperative surgical outcomes were collected and recorded before and after neoadjuvant therapy. Disease-free survival (DFS) and overall survival (OS) were used as long-term endpoints to evaluate the impact of pathological response on patient prognosis.

**Results:**

A total of 115 patients with LA-ESCC who underwent radical surgery after neoadjuvant camrelizumab combined with chemotherapy were finally included. Among them, 106 patients (92.2%) experienced treatment-related adverse events, of which only 16 cases were grade 3 or higher. Major pathological response (MPR) was achieved in 82 patients, with an MPR rate of 71.3%. The median follow-up duration was 43 months. Neither median overall survival (mOS) nor median disease-free survival (mDFS) was reached in the MPR group, while mOS and mDFS in the non-major pathological response (NMPR) group were 48 months and 41 months, respectively, indicating a significantly better prognosis in the MPR group than the NMPR group. The neutrophil-to-lymphocyte ratio (NLR) after neoadjuvant therapy was significantly different between patients with different pathological responses (P < 0.05).

**Conclusion:**

For patients with LA-ESCC, neoadjuvant camrelizumab combined with chemotherapy followed by radical surgery can significantly improve the rate of complete pathological response (CPR) and MPR, with a favorable overall safety profile. This study further confirms that lower NLR after neoadjuvant therapy is associated with better postoperative pathological remission, suggesting that NLR may serve as a convenient and readily available biomarker for predicting the efficacy of neoadjuvant immunochemotherapy in LA-ESCC.

## 1 Introduction

Esophageal cancer (EC) is a highly prevalent malignant tumor of the digestive system worldwide. In 2022, EC ranked as the seventh most commonly diagnosed cancer globally, with 604,000 new cases (accounting for 3.1% of all new cancer cases), and the sixth leading cause of cancer-related mortality, with 544,000 deaths (1, 2). Esophageal squamous cell carcinoma (ESCC) is the predominant pathological subtype of EC, accounting for more than 90% of all cases in China, resulting in an extremely high disease burden in the country (3).

Surgery, chemotherapy, radiotherapy, and immunotherapy constitute the mainstay of current multimodal treatments for EC. Although surgical resection remains the cornerstone of curative treatment for locally advanced esophageal squamous cell carcinoma (LA-ESCC), its long-term efficacy remains unsatisfactory, with a 5-year overall survival rate of less than 35% (4). In recent years, promising clinical outcomes have been reported with immune checkpoint inhibitors, particularly the anti-PD-1 antibody camrelizumab, in both advanced and perioperative settings of ESCC. For instance, the phase 3 ESCORT-NEO/NCCES01 trial and phase 2 NIC-ESCC2019 trial demonstrated that neoadjuvant camrelizumab combined with chemotherapy yielded a higher rate of complete pathological response (CPR) and major pathological response (MPR) without increasing the incidence of severe adverse events, supporting the safety and efficacy of this regimen in resectable LA-ESCC (5–7).

However, previous studies have notable limitations, including relatively short follow-up periods and a lack of long-term survival data. The factors influencing clinical outcomes in LA-ESCC patients treated with neoadjuvant camrelizumab plus chemotherapy remain incompletely elucidated. Therefore, this study was designed to address this research gap, while comprehensively evaluating the safety profile of this neoadjuvant regimen in a real-world clinical setting.

## 2 Methods

### 2.1 Study population

A retrospective analysis was performed on consecutive patients with LA-ESCC treated at the First Affiliated Hospital of Zhengzhou University between November 2019 and September 2024. A total of 115 patients were finally included after the application of inclusion and exclusion criteria. Peripheral blood parameters and relevant clinical characteristics were collected at baseline (before treatment initiation) and after 2 cycles of neoadjuvant camrelizumab combined with chemotherapy. The cutoff date for follow-up was November 30, 2025. This study was approved by the Ethics Committee of the First Affiliated Hospital of Zhengzhou University (Approval No.: 2024-KY-2025-002).

### 2.2 Inclusion criteria

Eligible patients met all the following criteria: (I) histologically confirmed LA-ESCC, with clinical stage cT2-4N0M0 or cTxN1-3M0; (II) aged between 18 and 80 years old; (III) Eastern Cooperative Oncology Group (ECOG) performance status score of 0 or 1; (IV) estimated life expectancy of more than 3 months; (V) adequate hematological, renal, and hepatic function; (VI) received 2 full cycles of neoadjuvant camrelizumab combined with chemotherapy, with complete clinical, laboratory, and imaging data before and after neoadjuvant treatment; (VII) underwent radical R0 resection after neoadjuvant therapy.

### 2.3 Exclusion criteria

Patients were excluded if they met any of the following criteria: (I) a history of hypersensitivity to camrelizumab, docetaxel, nedaplatin, nab-paclitaxel, or cisplatin; (II) current or previous autoimmune disease; (III) received immunosuppressive therapy or systemic corticosteroid therapy before enrollment; (IV) concurrent or previous other systemic malignant tumors; (V) severe cardiovascular, cerebrovascular, or renal diseases; (VI) stage IVB disease with distant organ metastasis, precluding curative surgery; (VII) received immune checkpoint inhibitors other than camrelizumab; (VIII) did not complete 2 full cycles of neoadjuvant camrelizumab combined with chemotherapy; (IX) lost to follow-up or with incomplete clinical data.

### 2.4 Treatment regimens

All included patients received first-line neoadjuvant camrelizumab combined with chemotherapy, with one of the following two regimens (1): Nab-paclitaxel-based regimen: camrelizumab (200 mg, day 1, every 3 weeks) + nab-paclitaxel (125 mg/m², day 1 and day 8, every 3 weeks) + cisplatin (75 mg/m², day 1, every 3 weeks) (2); Docetaxel-based regimen: camrelizumab (200 mg, day 1, every 3 weeks) + docetaxel (75 mg/m², day 1, every 3 weeks) + nedaplatin (75 mg/m², day 1, every 3 weeks).

### 2.5 Clinical and laboratory parameters

Baseline clinical data and peripheral blood laboratory parameters were collected for all patients. General clinical characteristics included age, gender, height, weight, ECOG score, clinical TNM stage, smoking history, drinking history, and treatment regimen. Hematological, inflammatory, and nutritional indices included neutrophil-to-lymphocyte ratio (NLR), platelet-to-lymphocyte ratio (PLR), lymphocyte-to-monocyte ratio (LMR), systemic immune-inflammation index (SII), prognostic nutritional index (PNI), and geriatric nutritional risk index (GNRI).

### 2.6 Efficacy assessment

#### 2.6.1 Pathological response evaluation

Postoperative pathological regression was graded according to the Becker tumor regression grading system (8), which assesses the proportion of residual viable tumor cells relative to the total tumor bed. The Becker system is classified into three grades: Grade 1 (complete or subtotal regression, with 0% or <10% residual viable tumor cells), Grade 2 (partial regression, with 10–50% residual viable tumor cells), and Grade 3 (minimal or no regression, with >50% residual viable tumor cells). For statistical analysis, patients with Becker Grade 1 were categorized into the MPR group, while those with Becker Grade 2 and 3 were categorized into the NMPR group. CPR was defined as 0% residual viable tumor cells (Becker Grade 1a).

#### 2.6.2 Safety assessment

Treatment-related adverse events during neoadjuvant therapy were recorded and graded according to the National Cancer Institute Common Terminology Criteria for Adverse Events (CTCAE) Version 4.0 (9), Perioperative safety was evaluated by collecting data on operation duration, number of dissected lymph nodes, postoperative hospital stay, and postoperative complications.

#### 2.6.3 Survival follow-up

Patients were followed up via telephone interviews, outpatient medical records, and inpatient hospitalization data. The primary long-term endpoints were DFS and OS. DFS was defined as the time from radical surgery to the first event of tumor recurrence, disease progression, or death from any cause. OS was defined as the time from the initiation of neoadjuvant immunochemotherapy to death from any cause.

### 2.7 Statistical analysis

All statistical analyzes were performed using SPSS 26.0 (IBM Corp, Armonk, NY, USA), and R 4.4.3 (R Foundation for Statistical Computing, Vienna, Austria).

Continuous variables with a normal distribution were presented as mean ± standard deviation (SD), and intergroup comparisons were performed using the independent samples t-test. Non-normally distributed continuous variables were reported as median and interquartile range (IQR, M [P25, P75]), and the Mann-Whitney U test was used for between-group comparisons. Categorical variables were expressed as counts and percentages [n (%)], and intergroup differences were analyzed using the chi-square (χ²) test.

The Kaplan-Meier method was applied to estimate disease-free survival (DFS) and overall survival (OS) in the major pathological response (MPR) and non-major pathological response (NMPR) subgroups, and the two-sided log-rank test was used to compare survival curves between groups. Visualization of survival curves and box-and-whisker plots for post-neoadjuvant therapy neutrophil-to-lymphocyte ratio (NLR) levels was performed using R 4.4.3 with the ggplot2, ggpubr, and ggsci packages.

A two-sided P-value < 0.05 was considered statistically significant.

## 3 Results

**Figure 1:**
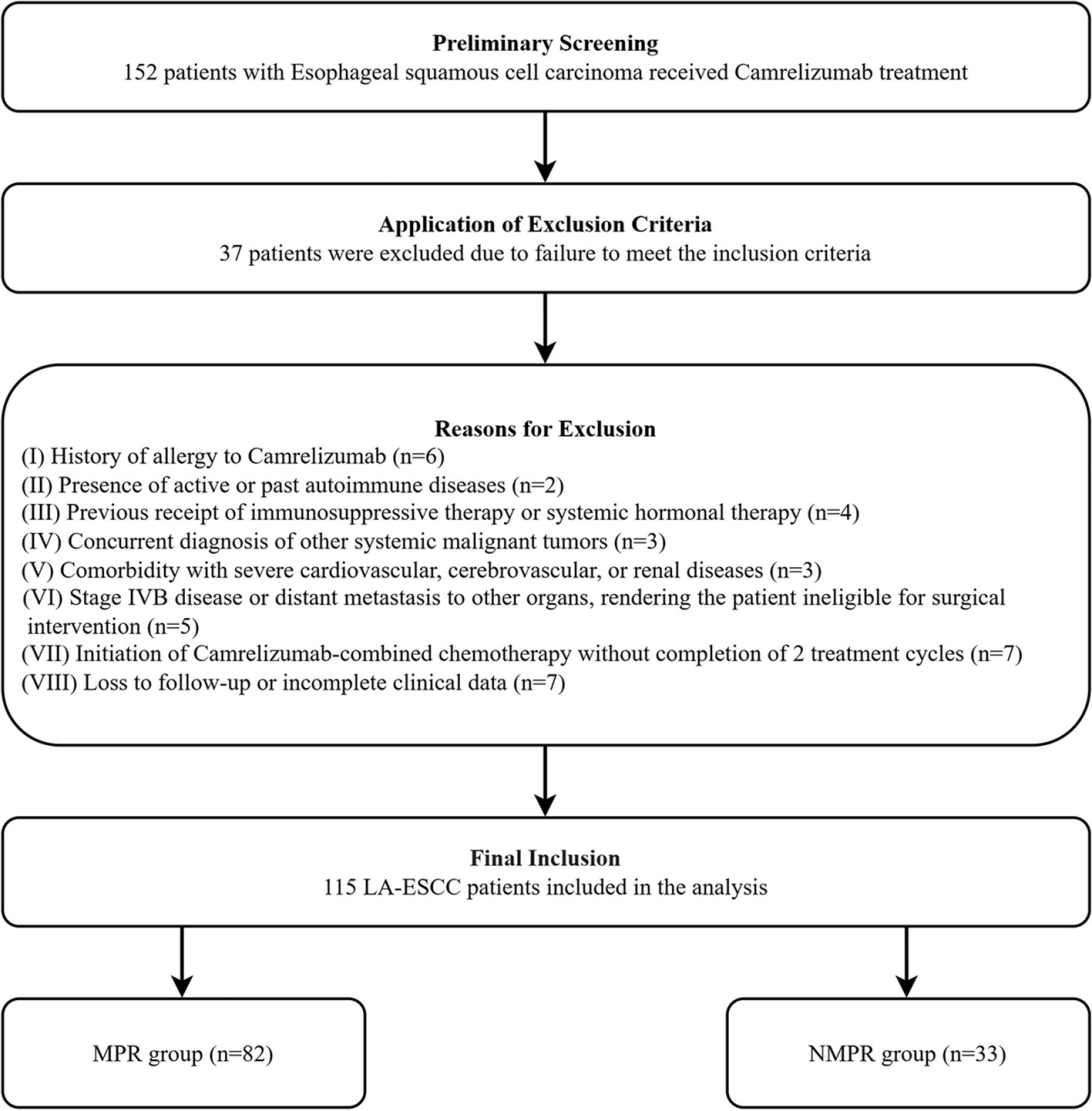
Flowchart of the patient selection process.

A total of 152 consecutive patients with LA-ESCC who received neoadjuvant camrelizumab-based immunochemotherapy were initially screened, and 115 patients were finally included in the analysis after applying the exclusion criteria (**Figure 1**).

### 3.1 Baseline clinical characteristics of patients

Among the 115 included patients with LA-ESCC, 43 patients were older than 65 years, and 72 patients were 65 years or younger. 47 patients received the docetaxel plus nedaplatin-based regimen, while 68 received the Nab-paclitaxel plus cisplatin-based regimen. Regarding clinical stage, 21 patients (18.3%) were stage II, 41 patients (35.7%) were stage III, and 53 patients (46.1%) were stage IVA (**Table 1**).

**Table 1:** Baseline characteristics of the study population.

Characteristics	Total (n=115)	MRP (n=82)	NMRP (n=33)	P
Age, n (%)				0.257
≤65	72 (62.6%)	54 (65.9%)	18 (54.5%)	
>65	43 (37.4%)	28 (34.1%)	15 (45.5%)	
Gender, n (%)				0.984
Male	80 (69.6%)	57 (69.5%)	23 (69.7%)	
Female	35 (30.4%)	25 (30.5%)	10 (30.3%)	
ECOG PS, n (%)				0.163
0	65 (56.5%)	49 (59.8%)	16 (48.5%)	
1	50 (43.5%)	33 (40.2%)	17 (51.5%)	
Smoking history, n (%)				0.685
No	73 (63.5%)	53 (64.6%)	20 (60.6%)	
Yes	42 (36.5%)	29 (35.4%)	13 (39.4%)	
Alcohol consumption, n (%)				0.110
No	69 (60.0%)	53 (64.6%)	16 (48.5%)	
Yes	46 (40.0%)	29 (35.4%)	17 (51.5%)	
Tumor location, n (%)				0.212
upper	17 (14.8%)	9 (11.0%)	8 (24.2%)	
middle	45 (39.1%)	34 (41.5%)	11 (33.3%)	
Lower	53 (46.1%)	39 (47.6%)	14 (42.4%)	
cTNM stage, n (%)				0.289
II	21 (18.3%)	16 (19.5%)	5 (15.2%)	
III	41 (35.7%)	32 (39.0%)	9 (27.3%)	
IVA	53 (46.1%)	34 (41.5%)	19 (57.6%)	
Treatment Regimen, n (%)				0.144
Nab-Paclitaxel-based regimen	68 (59.1%)	45 (54.9%)	23 (69.7%)	
Docetaxel-based regimen	47 (40.9%)	37 (45.1%)	10 (30.3%)	

ECOG PS, Eastern Cooperative Oncology Group Performance Status; cTNM, clinical Tumor-Node-Metastasis; BMI, body mass index; kg, kilogram;.

### 3.2 Postoperative pathological response

**Figure 2:**
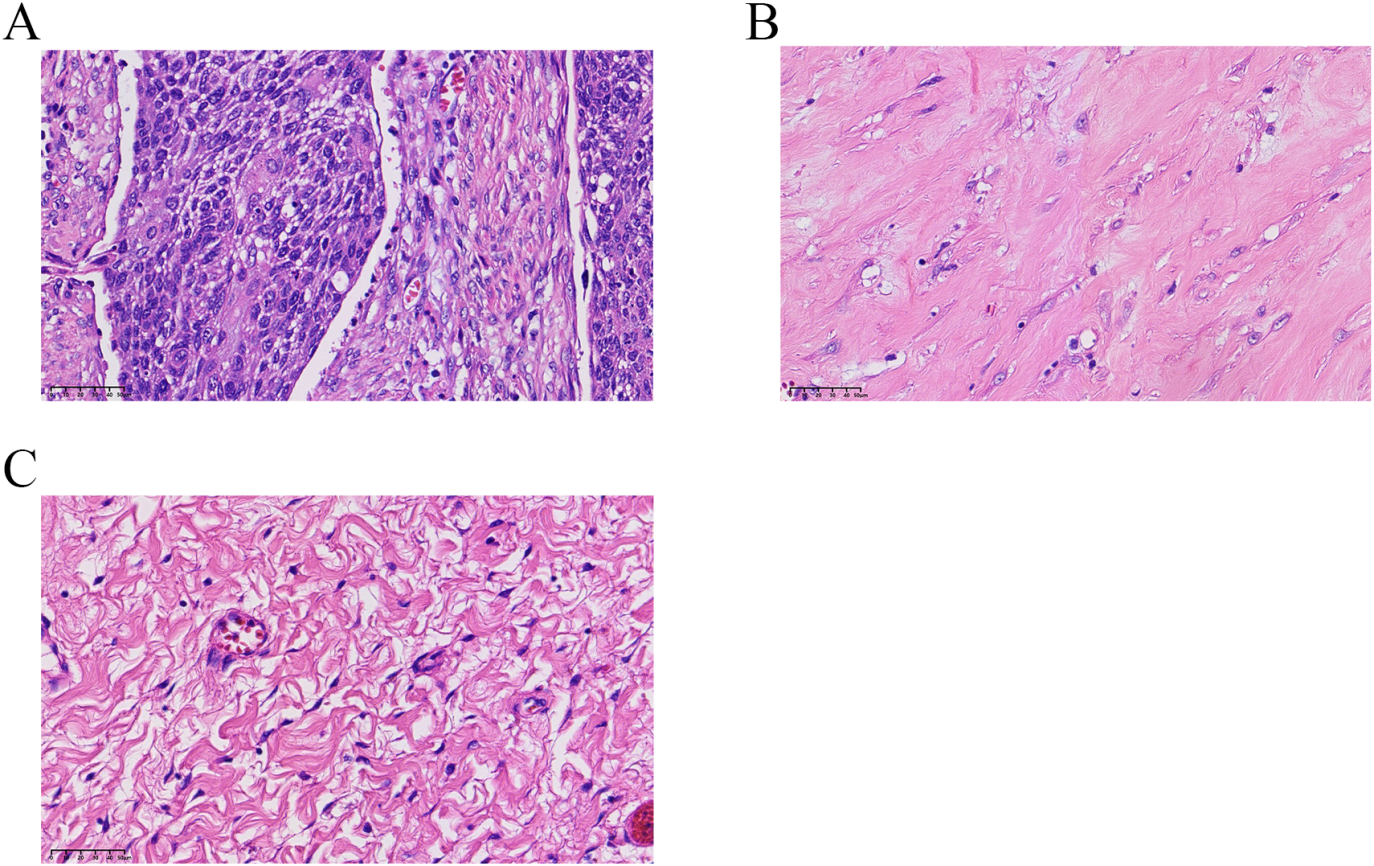
Pathological response of esophageal squamous cell carcinoma after neoadjuvant therapy (hematoxylin and eosin staining, original magnification ×40). **(A)** Non-major pathological response (NMPR): Dense nests of viable residual esophageal squamous cell carcinoma cells with nuclear atypia are observed in the tumor bed, corresponding to ≥10% residual viable tumor. **(B)** Major pathological response (MPR): Scattered foci of residual viable tumor cells are visible within an extensive background of stromal fibrosis and inflammatory infiltration, corresponding to <10% residual viable tumor. **(C)** Complete pathological response (CPR): No viable residual tumor cells are identified in the tumor bed, with only acellular fibrous stroma, hyalinization, and scattered inflammatory cells observed, corresponding to 0% residual tumor.

All 115 patients achieved R0 radical resection after neoadjuvant camrelizumab combined with chemotherapy. Among them, 32 patients (27.8%) achieved CPR (Becker Grade 1a), and 50 patients (43.5%) achieved subtotal regression (Becker Grade 1b). Overall, MPR was achieved in 82 patients, with an MPR rate of 71.3%. Representative histopathological images of tumor specimens from patients with NMPR, MPR, and CPR are shown in **Figure 2**.

### 3.3 Safety assessment of neoadjuvant therapy

During neoadjuvant treatment, 106 patients (92.2%) experienced treatment-related adverse events. The majority of adverse events were grade 1 or 2, most commonly myelosuppression, followed by thyroid dysfunction, liver function impairment, electrolyte abnormalities, and reactive cutaneous capillary endothelial proliferation (RCCEP). Grade ≥3 adverse events occurred in 16 patients (13.9%), including leukopenia in 11 cases, electrolyte abnormalities in 3 cases, elevated creatinine in 1 case, and nausea and vomiting in 1 case (**Table 2**).

**Table 2:** Treatment-related adverse events during neoadjuvant therapy.

Drug-related adverse reactions	Grade 1–2, n (%)	Grade≥3, n (%)
Any adverse reaction	106 (92.2%)	16 (13.9%)
Anemia	45 (39.1%)	0
Leukopenia	33 (28.7%)	12 (10.4%)
Neutropenia	30 (26.1%)	11 (9.6%)
Thrombocytopenia	16 (13.9%)	0
Thyroid dysfunction	22 (19.1%)	0
Creatinine increased	6 (5.2%)	1 (0.9%)
Transaminase increased	10 (8.7%)	0
Nausea, vomiting	12 (10.4%)	1 (0.9%)
Diarrhea	6 (5.2%)	0
Fatigue	11 (9.6%)	0
Electrolyte abnormalities	23 (20%)	3 (2.6%)
RCCEP	9 (7.8%)	0

TRAEs, treatment-related adverse events; RCCEP, Reactive cutaneous capillary endothelial cell proliferation.

### 3.4 Perioperative surgical safety

All 115 patients underwent radical surgery after neoadjuvant therapy, with a 100% R0 resection rate. The mean operation duration was 5.15 ± 1.32 hours. The median number of dissected lymph nodes was 34 (range: 28–75). The median postoperative hospital stay was 9 days (range: 8–11 days). The 30-day mortality was 0.9% (1 case), and the 90-day mortality was 2.6% (3 cases). Postoperative complications occurred in 30 patients (26.1%), mainly pulmonary infection (18 cases, 15.7%) and recurrent laryngeal nerve injury (8 cases, 7.0%), followed by anastomotic leakage (4 cases, 3.5%), pleural effusion (4 cases, 3.5%), and respiratory failure (2 cases, 1.7%). Most grade ≥3 postoperative complications were anastomotic leakage and respiratory failure (**Tables 3**, **4**).

**Table 3:** Perioperative surgical safety outcomes.

Variables	MPR (n=82)	NMPR (n=33)
Operation time, h	4.78 ± 1.23	5.27 ± 1.33
Number of lymph nodes harvested	33 (26.8-43.3)	36 (29.5-43.5)
Postoperative hospital stay, d	8.5 (8–10)	10 (7.0-12.5)
30 days Mortality, n (%)	1 (0.9%)	0
90 days Mortality, n (%)	2 (1.7%)	1 (0.9%)

h, hour; n, number; d, day; %, percentage.

**Table 4:** Incidence of postoperative complications.

Variables	Grade 1–2, n (%)	Grade≥3, n (%)
Any adverse reaction	30 (26.1%)	3 (2.6%)
Pulmonary infection	18 (15.7%)	0
Recurrent laryngeal nerve injury	8 (7.0)	0
Anastomotic leakage	4 (3.5%)	2 (1.7%)
Pleural effusion	4 (3.5%)	1 (0.9%)
Respiratory failure	2	2

n, number; %, percentage.

### 3.5 Comparison of clinical and laboratory parameters between groups

**Figure 3:**
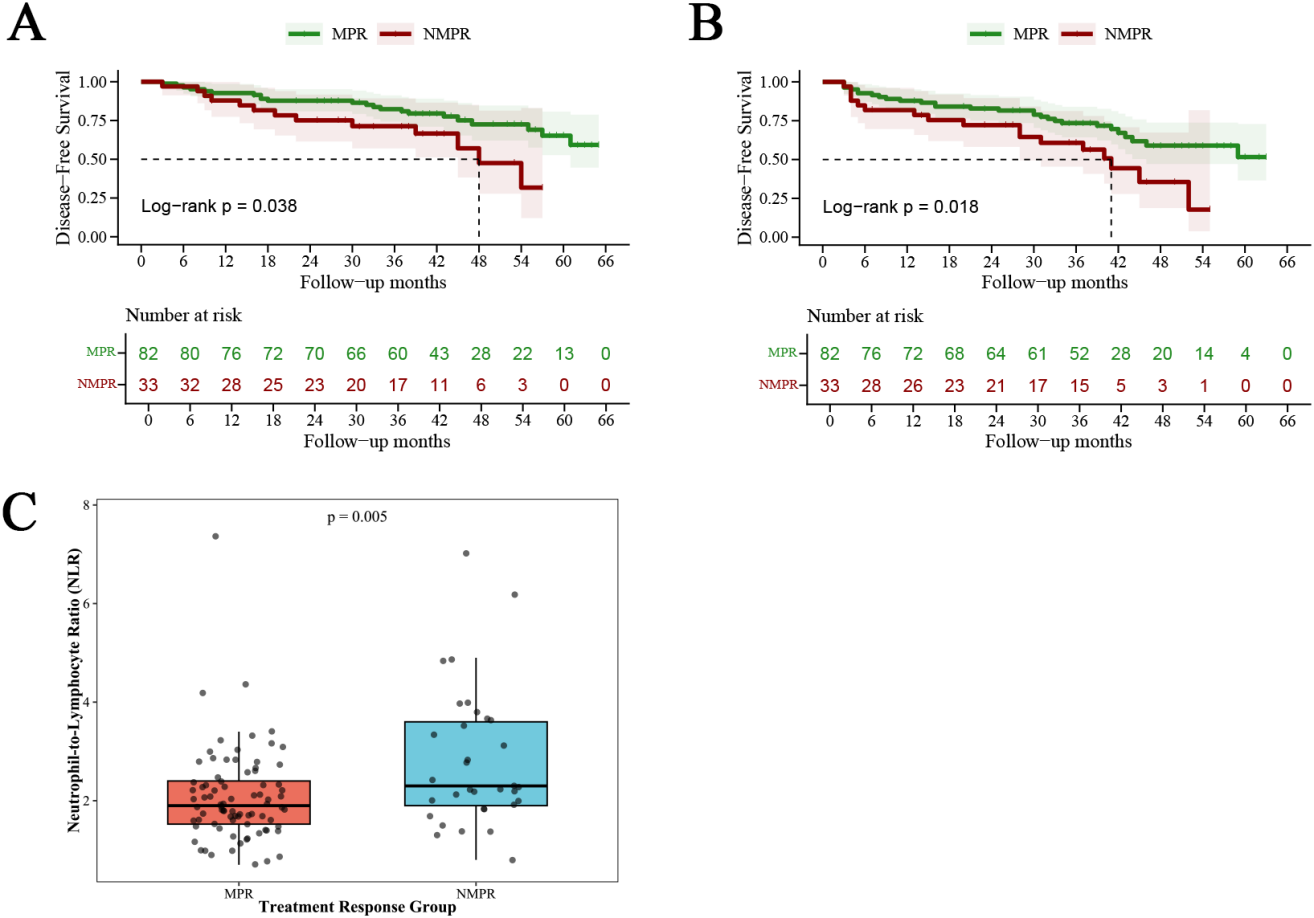
Major pathological response correlates with improved survival and lower post-neoadjuvant therapy neutrophil-to-lymphocyte ratio. **(A, B)** Kaplan-Meier curves for disease-free survival [**(A)**, P = 0.038] and overall survival [**(B)**, P = 0.018] in MPR and non-MPR groups, with number-at-risk tables included below each curve. **(C)** Box-and-whisker plot of post-neoadjuvant therapy Neutrophil-to-Lymphocyte Ratio (NLR) levels in MPR and NMPR groups (P = 0.005).

There were no significant differences in baseline clinical characteristics between the MPR and NMPR groups (all P > 0.05, **Table 1**). After neoadjuvant therapy, the NLR was significantly lower in the MPR group than the NMPR group (P < 0.05, **Figure 3C**). No significant differences were observed in other baseline or post-treatment hematological, inflammatory, and nutritional parameters between the two groups (all P > 0.05, **Tables 5**, **6**).

**Table 5:** Comparison of baseline hematological, inflammatory, and nutritional parameters between MPR and NMPR groups.

Variables	MPR (n=82)	NMPR (n=33)	P
BMI, kg/m²	23.87 ± 0.68	22.91 ± 0.33	0.163
Hemoglobin, g/L	133.4 (119.8-145.4)	136.0 (124–146)	0.449
White blood cell, ×10^9^/L	6.07 (5.07-7.03)	6.37 (5.61-6.75)	0.880
Platelets, ×10^9^/L	213.5 (187.3-253.3)	213 (167.5-248.5)	0.420
Neutrophil, ×10^9^/L	3.7 (2.90-4.57)	3.85 (2.97-4.63)	0.848
Lymphocyte, ×10^9^/L	1.63 (1.39-1.97)	1.73 (1.17-2.14)	0.880
Monocyte, ×10^9^/L	0.44 (0.36-0.53)	0.41 (0.33-0.5)	0.392
NLR	2.32 (1.72-2.93)	2.03 (1.51-3.58)	0.767
PLR	131.1 (106.4-164.9)	118 (104.7-174.9)	0.608
LMR	3.98 ± 1.38	4.35 ± 1.76	0.251
SII	488.9 (372.1-653.3)	44.2 (288.0-709.6)	0.540
PNI	49.2 (46.5-52.5)	48.6 (44.4-53.0)	0.704
GNRI	103.79 ± 8.7	103.70 ± 9.76	0.965

BMI, body mass index; kg, kilogram; m, meter; g/L, gram per liter; ×10^9^/L, ×10^9^ per liter; NLR, neutrophil-to-lymphocyte ratio; PLR, platelet-to-lymphocyte ratio; LMR, lymphocyte-to-monocyte ratio; SII, systemic immune-inflammation index; PNI, prognostic nutritional index; GNRI, geriatric nutritional risk index.

**Table 6:** Comparison of post-neoadjuvant therapy hematological, inflammatory, and nutritional parameters between MPR and NMPR groups.

Variables	MPR (n=82), n (%)	NMPR (n=33), n (%)	P
Hemoglobin, g/L	112 (103.2-121.2)	113.1 (106-123)	0.612
White blood cell, ×10^9^/L	5.9 (5.0-7.4)	5.8 (4.8-7.2)	0.619
Platelet, ×10^9^/L	202 (167-237)	193.5 (258.8-236.3)	0.683
Neutrophil, ×10^9^/L	3.5 (2.9-4.65)	3.4 (2.6-4.5)	0.485
Lymphocyte, ×10^9^/L	1.65 ± 0.55	1.64 ± 0.51	0.935
Monocyte, ×10^9^/L	0.4 (0.4-0.5)	0.5 (0.4-0.6)	0.330
NLR	1.9 (1.5-2.43)	2.3 (1.85-3.65)	0.005
PLR	124 (99.7-156.3)	126.4 (101.0-157.0)	0.814
LMR	3.9 (2.9-439)	3.6 (2.68-4.63)	0.518
SII	466.5 (357.3-627.8)	422.5 (275.4-590.1)	0.311
PNI	48.1 (15.4-50.5)	47.85 (44.48-50.92)	0.736
GNRI	101.3 ± 7.3	101.8 ± 8.21	0.762

BMI, body mass index; kg, kilogram; m, meter; g/L, gram per liter; ×10^9^/L, ×10^9^ per liter; NLR, neutrophil-to-lymphocyte ratio; PLR, platelet-to-lymphocyte ratio; LMR, lymphocyte-to-monocyte ratio; SII, systemic immune-inflammation index; PNI, prognostic nutritional index; GNRI, geriatric nutritional risk index.

### 3.6 Survival outcomes between the MPR and NMPR groups

The median follow-up duration was 43 months. Kaplan-Meier survival analysis showed that patients in the MPR group had significantly better OS and DFS than those in the NMPR group (log-rank P = 0.038 for OS, P = 0.018 for DFS). Neither mOS nor mDFS was reached in the MPR group during the follow-up period, while mOS and mDFS in the NMPR group were 48 months and 41 months, respectively (**Figures 3A, B**).

## 4 Discussion

Esophageal squamous cell carcinoma (ESCC) remains the dominant histological subtype of esophageal cancer in China, with locally advanced disease associated with poor long-term survival despite radical surgery (2, 10). In recent years, immune checkpoint inhibitors have revolutionized the multidisciplinary management of ESCC, and the programmed cell death protein 1 (PD-1) inhibitor camrelizumab has emerged as a cornerstone agent in both advanced and resectable disease settings. Previous phase 3 trial data from the ESCORT-1st study first established the survival benefit of first-line camrelizumab plus chemotherapy over chemotherapy alone in advanced ESCC, significantly prolonging overall survival (OS) with a manageable safety profile (11). Subsequent studies further validated the antitumor efficacy of camrelizumab in the neoadjuvant setting across multiple solid tumors, including breast cancer, hepatocellular carcinoma, cervical cancer, and locally advanced ESCC (LA-ESCC) (12–15). Specifically for resectable LA-ESCC, accumulating evidence from phase 2 and 3 trials has demonstrated that neoadjuvant camrelizumab combined with chemotherapy achieves encouraging objective response rates of 74% to 90.5%, a high rate of radical R0 resection, and a low incidence of grade ≥3 treatment-related adverse events (TRAEs) (16, 17). Building on this robust clinical evidence, we designed this retrospective cohort study to further validate the efficacy and safety of this neoadjuvant regimen in a real-world LA-ESCC population, and to explore potential predictive biomarkers for treatment response.

In our cohort, neoadjuvant camrelizumab combined with chemotherapy followed by radical surgery exhibited a favorable overall tolerability profile. Consistent with previous reports, the overall incidence of TRAEs was 92.2% in our study, the vast majority of which were mild to moderate (grade 1–2) hematologic toxicities, predominantly myelosuppression. These events were clinically manageable, and nearly all patients recovered with appropriate supportive care, with no treatment-related deaths reported during the neoadjuvant phase. Notably, all enrolled patients successfully completed the planned neoadjuvant regimen and underwent curative-intent radical R0 esophagectomy, achieving a 100% R0 resection rate. When compared with the surgical outcomes from the pivotal phase 3 ESCORT-NEO/NCCES01 trial (6), we found no significant differences in key perioperative metrics including operative duration, number of harvested lymph nodes, incidence of postoperative complications, and 30- and 90-day all-cause mortality. These findings further corroborate the safety and feasibility of neoadjuvant camrelizumab-based chemoimmunotherapy in real-world clinical practice for resectable LA-ESCC. The primary goal of neoadjuvant therapy for resectable LA-ESCC is to achieve deep pathological response, which has been consistently linked to improved long-term survival (20, 21).

In recent years, landmark global phase 3 trials including KEYNOTE-590 and CheckMate-648 have established the standard role of immune checkpoint inhibitors in the first-line treatment of advanced ESCC (18, 19), and this benefit has been extended to the neoadjuvant setting for resectable disease. Previous studies have reported that neoadjuvant immunochemotherapy (nICT) yields a complete pathological response (CPR) rate of over 20% and a major pathological response (MPR) rate exceeding 40% in LA-ESCC patients (7), while specialized studies of neoadjuvant camrelizumab-based chemoimmunotherapy have reported even more promising efficacy, with a CPR rate up to 39.6% and an MPR rate as high as 77.1% (20). In our cohort, we observed an MPR rate of 71.3% and a CPR rate of 27.8%, which are highly aligned with these published real-world and clinical trial data, further validating the robust pathological response efficacy of this neoadjuvant regimen.

Major pathological response (MPR), defined as ≤10% residual viable tumor cells in the surgical specimen after neoadjuvant therapy as per our pre-specified Becker tumor regression grading system, has emerged as a critical intermediate efficacy endpoint for neoadjuvant therapy in solid tumors. This standardized definition has been widely validated and adopted for pathological response assessment across multiple malignancies, most extensively in non-small cell lung cancer (NSCLC) (21). Fundamentally, MPR reflects a high degree of tumor chemosensitivity and immunosensitivity to neoadjuvant treatment, and is increasingly recognized as a core biomarker for evaluating the success of neoadjuvant regimens. Accumulating clinical evidence has established that patients who achieve MPR have consistently superior long-term survival outcomes, and MPR is regarded as a robust surrogate endpoint for overall survival (OS) in patients receiving neoadjuvant therapy (22, 23). Multiple large-scale cohort studies have consistently demonstrated that MPR is associated with significantly improved OS and disease-free survival (DFS) across various solid tumors, compared with non-major pathological response (NMPR) (24). However, the majority of existing research on the prognostic value of MPR has been conducted in lung cancer populations, with relatively limited data in esophageal squamous cell carcinoma (ESCC), and findings from lung cancer research provide a critical methodological and clinical reference for ESCC studies. Consistent with these established findings, our study revealed that LA-ESCC patients who achieved MPR had significantly better DFS and OS than those with NMPR. This finding further validates that MPR is a reliable surrogate endpoint for neoadjuvant chemoimmunotherapy efficacy, and can serve as an early predictive marker of long-term survival benefit in LA-ESCC patients.

Chronic inflammation is a well-established hallmark of cancer, and plays a complex, multifaceted role in modulating the tumor immune microenvironment (TIME) and tumor progression. The TIME is extensively populated by a diverse array of innate and adaptive inflammatory cells, which dynamically regulate tumor growth, invasion, and metastasis through the release of both pro-tumorigenic and anti-tumorigenic soluble mediators (25). For example, tumor-associated macrophages (TAMs) and tumor-infiltrating neutrophils commonly polarize to an immunosuppressive, tumor-promoting phenotype in the TIME, driving extracellular matrix remodeling, angiogenesis, and metastatic dissemination of tumor cells. Given the close crosstalk between systemic inflammation and the TIME, peripheral blood inflammatory biomarkers have emerged as non-invasive, readily accessible tools for predicting immunotherapy response in clinical practice. A growing body of evidence has demonstrated that a panel of systemic inflammatory and nutritional biomarkers, including the neutrophil-to-lymphocyte ratio (NLR), platelet-to-lymphocyte ratio (PLR), systemic immune-inflammation index (SII), and prognostic nutritional index (PNI), are reliable predictors of immunotherapy efficacy across multiple solid malignancies, including small cell lung cancer, breast cancer, and gastric cancer (26–28).

The neutrophil-to-lymphocyte ratio (NLR), a readily available, non-invasive biomarker reflecting systemic inflammatory status, has been widely validated as a critical prognostic indicator in patients with esophageal squamous cell carcinoma (ESCC). Consistent with our findings, high pretreatment NLR values are consistently linked to an immunosuppressive tumor microenvironment, inferior treatment response, and poor survival outcomes in ESCC patients (28). A comprehensive meta-analysis by Wu et al. (29), which pooled data from 18 studies including 6119 ESCC patients, confirmed that elevated NLR was significantly associated with worse clinical outcomes, with hazard ratios of 1.47 for overall survival (OS), 1.62 for disease-free survival (DFS), and 1.62 for progression-free survival, respectively, in patients with high versus low NLR.

In our cohort, we found that LA-ESCC patients who achieved major pathological response (MPR) after neoadjuvant camrelizumab-based chemoimmunotherapy had significantly lower post-neoadjuvant NLR levels than those with non-major pathological response (NMPR) (P = 0.005). This key finding suggests that dynamic reduction in NLR after neoadjuvant therapy is a strong predictor of favorable pathological response, which further mediates the superior long-term OS and DFS observed in the MPR group compared with the NMPR group in our study. This finding also supports the potential of post-neoadjuvant NLR as an early on-treatment biomarker to stratify patients who may derive maximum benefit from camrelizumab-based neoadjuvant chemoimmunotherapy.

However, we did not detect a significant association between baseline NLR and pathological response or long-term survival in our LA-ESCC cohort, which is inconsistent with some previous dynamic biomarker studies in ESCC. Ferrucci et al. (30) reported that low baseline NLR was associated with significantly prolonged OS in LA-ESCC patients receiving immunotherapy. Several factors may explain this discrepancy: first, our study is a single-center retrospective cohort with a relatively small sample size, which may limit the statistical power to detect the prognostic effect of baseline NLR; second, the heterogeneity of neoadjuvant chemotherapy regimens, baseline patient characteristics, and follow-up duration across studies may also contribute to the inconsistent findings. The prognostic value of baseline versus dynamic changes in NLR for neoadjuvant chemoimmunotherapy in LA-ESCC warrants further validation in large-scale, multicenter, prospective cohorts.

In conclusion, our real-world retrospective cohort study demonstrates that neoadjuvant camrelizumab-based chemoimmunotherapy followed by radical R0 esophagectomy is a safe, feasible, and effective treatment strategy for patients with resectable locally advanced esophageal squamous cell carcinoma (LA-ESCC). This regimen achieves a 100% R0 resection rate with a manageable safety profile, and yields encouraging pathological response outcomes, with an MPR rate of 71.3% and a CPR rate of 27.8%. More importantly, achieving MPR is significantly associated with superior long-term OS and DFS, supporting MPR as a reliable surrogate endpoint for neoadjuvant treatment efficacy in LA-ESCC. Furthermore, we identified that post-neoadjuvant NLR, a routinely tested non-invasive peripheral blood biomarker, is significantly correlated with postoperative pathological response, which may serve as a convenient on-treatment marker for early prediction of neoadjuvant chemoimmunotherapy efficacy in LA-ESCC patients.

Nevertheless, several limitations of this study should be acknowledged. First, this was a single-center retrospective study conducted at a high-volume tertiary hospital in China, with inherent selection bias and confounding factors associated with retrospective study design. Second, all patients received camrelizumab as the sole immune checkpoint inhibitor, with no enrollment of patients treated with other PD-1/PD-L1 inhibitors (including sintilimab, toripalimab, or tislelizumab). Therefore, the generalizability of our findings to other immune checkpoint inhibitor-based neoadjuvant regimens is limited. Third, although the median follow-up duration reached 43 months, the median OS and DFS for the overall cohort were not reached at the final data cutoff, meaning that extended follow-up is needed to validate the mature long-term survival benefits and the long-term prognostic value of post-neoadjuvant NLR.

In light of these limitations, a multicenter, large-scale prospective clinical trial is required to further validate the efficacy, safety, and predictive biomarkers identified in our study. In addition, future studies should focus on exploring more sensitive, specific, and non-invasive biomarkers to optimize patient stratification, guide personalized neoadjuvant treatment selection, and ultimately improve the long-term clinical outcomes of patients with LA-ESCC.

## 5 Conclusion

For patients with LA-ESCC, neoadjuvant camrelizumab combined with chemotherapy followed by radical surgery can significantly improve the rate of CPR and MPR, with a favorable overall safety profile. This study further confirms that a lower NLR after neoadjuvant therapy is associated with better postoperative pathological remission, suggesting that this index can serve as a convenient biomarker for predicting the efficacy of neoadjuvant immunochemotherapy in LA-ESCC.

## Data Availability

The data analyzed in this study is subject to the following licenses/restrictions: Data will be made available on request. Requests to access these datasets should be directed to JP, pjx2745644495@163.com.
